# Pre-operative axillary staging: should core biopsy be preferred to fine needle aspiration cytology?

**DOI:** 10.3332/ecancer.2017.724

**Published:** 2017-03-07

**Authors:** Raghavan Vidya, Fahad Mujtaba Iqbal, Bernadette Bickley

**Affiliations:** 1 County Hospital, Weston Road, Stafford ST16 3SA, UK; 2 Keele University, David Weatherall Building, Keele University, Stoke-on-Trent, Staffordshire ST5 5BG, UK; 3 County Hospital, Weston Road, Stafford ST16 3SA, UK

**Keywords:** preoperative staging, sensitivity, core needle, fine needle aspiration, breast

## Abstract

**Objective:**

To determine the diagnostic accuracy of ultrasound guided fine needle aspiration (FNA) cytology and core needle biopsy (CNB) of axillary lymph nodes pre-operatively in newly diagnosed operable primary breast cancer.

**Methods:**

An observational study for all patients who underwent pre-operative FNA cytology or CNB during September 2013–August 2014 was conducted at our institution (County Hospital, Stafford, UK). The accuracy of pre-operative axillary staging was compared to the post-operative histology. For this sensitivity, specificity, positive predictive value (PPV) and negative predictive values (NPV) were calculated.

**Results:**

A total of 81 consecutive patients were evaluated by axillary ultrasound. Patients identified with potentially abnormal axillary lymph nodes underwent definitive surgery. Seven patients had positive cytology/histology who did not undergo definitive surgery and were excluded (N = 74) from the study. CNB had a sensitivity of 100% versus 72% (p = 0.006) for FNA cytology. Both had 100% specificity and PPV. The NPV of CNB was 100% versus 72% for FNA cytology. Among 35% of patients that underwent FNA cytology required repeat procedure versus 2.6% of patients who underwent CNB. 0/38 patients that had CNB required a second operation while 7/43 patients with negative FNA cytology had positive lymph nodes identified at sentinel lymph node biopsy (SLNB) requiring surgical re-intervention with axillary node clearance.

**Conclusion:**

CNB was superior to FNA cytology when interrogating the axilla. We recommend CNB to be adopted routinely in pre-operative axillary staging to reduce surgical re-intervention.

## Introduction

Lymph node status is one of the most significant prognostic factors for patients with breast cancer [1]. Sentinel lymph node biopsy (SLNB) has become the standard of care in predicting the status of remaining lymph nodes with surgical intervention as we see axillary lymph node clearance occurring in lymph node positive patients [2]. Axillary lymph node clearance negatively affects patient quality of life (QoL) with significant morbidity [3].

In order to eliminate unnecessary axillary lymph node clearance, preoperative axillary staging using ultrasound (US) is routinely performed in all newly diagnosed breast cancer patients with reported specificities of 100% [4]. Identification of metastatic spread to the lymph nodes prior to surgery using US-guided fine needle aspiration (FNA) cytology or core needle biopsy (CNB) is of great importance for accurate staging and for reducing the need for SLNB [5].

The aim of this study was to determine the diagnostic accuracy of US-guided FNA cytology and CNB of axillary lymph node pre-operatively in newly diagnosed operable primary breast cancer.

## Methods

This was approved by our local audit department. A total of 170 consecutive patients from September 2013–August 2014 with newly diagnosed breast cancer who had an axillary ultrasound (US) were reviewed. Abnormal or suspicious axillary lymph nodes were identified in 81 patients (47.6%) and were included in the study. Axillary US was normal in 89 patients (52.4%) who were excluded from the study.

An abnormal axillary node was biopsied using FNA cytology or CNB. Ultrasound-guided FNA cytology was performed utilising a 21-guage needle and CNB using a 14-guage automated biopsy device (Monopty, Bard Radiology, Covington, Georgia, USA). Abnormal axillary node on US was defined as: i) entirely hypoechoic node, ii) a node cortex >2 mm, iii) eccentric cortical lobulation, iv) a short-long axis ratio >0.5, v) a short axis >10 mm, or vi) peripheral nodal vascularity.

In cases with more than one suspicious node, FNA cytology or CNB was performed on the node demonstrating the most suspicious features. The radiologists performed FNA cytology when the lymph node was high lying, close to a blood vessel, or to the chest wall. Pre-operative biopsy reports were compared with the histology following surgery. Pre-operatively when lymph node biopsy was negative, the gold standard for sentinel node detection was the ‘combined technique’ which used both the blue dye and radioisotope injection. In patients who preoperatively had positive lymph nodes, standard axillary clearance was carried out. The histology reports were retrieved from a pathology lab computer system.

Statistical analysis was performed with GraphPad (GraphPad Software, La Jolla California, USA). The sensitivity, specificity, positive predictive value (PPV) and negative predictive values (NPV) were calculated. A Fisher’s exact test was calculated with p<0.05 defined as statistically significant.

## Results

**Figure 1.**
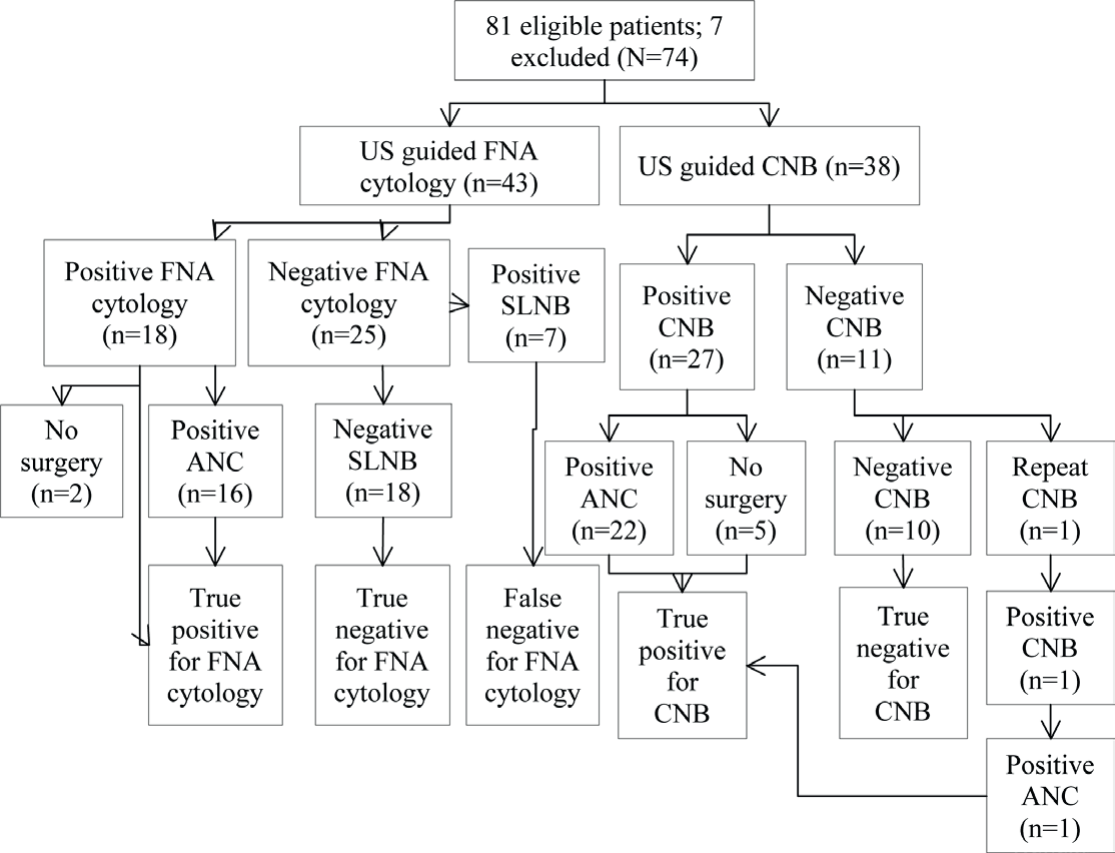
Flowchart of patients. US: ultrasound, CNB: core needle biopsy, FNA: fine needle aspiration, ANC: axillary lymph node clearance.

A total of 81 patients had preoperative biopsy (CNB, n = 38; FNA cytology, n = 43). The results are shown in Figure 1: seven patients had positive cytology/histology that did not undergo definitive surgery and hence were excluded from the analysis (N = 74).

43 patients (58.1 %) underwent FNA cytology of which 15 (34.8%) patients required a repeat procedure (n = 11 with technically inadequate cytology; n = 4 with suspicious cytology). In contrast, 38/74 (51.3%) patients underwent CNB with 1/38 (2.6%) requiring a repeat procedure (n = 1, a pathological node on US with benign histological changes demonstrating evidence of haemorrhage within the node, a repeat CNB because of the discordant result revealed malignant histological features).

True positives (TP) were cases with evidence of metastatic disease at CNB with metastatic deposits proven at axillary lymph node clearance (Figure 1, n = 27). True negatives (TN) were cases with negative histology for malignancy at CNB and were free of metastasis according to the gold standard SLNB (Figure 1, n = 11). False negatives (FN) had negative histology for malignancy at CNB but were found to have metastasis at SLNB. There were no false negatives in our study. False positives (FP) had positive histology at CNB but were free of metastasIs at axillary lymph node clearance, again, we had no FP (n = 0). Sensitivity (28 ÷ (28 + 0)), specificity, PPV, and NPV for CNB were 100% (Table 1).

TP were cases with evidence of metastatic disease identified on FNA positive cytology with metastatic deposits proven at axillary lymph node clearance (Figure 1, n = 18). TN were cases with negative cytology for malignancy at FNA cytology and were free of metastasis according to the gold standard SLNB (Figure 1, n = 25). FN were cases with negative cytology for malignancy at FNA cytology but had metastasis at SLNB (n = 7). These patients had surgical re-intervention as axillary lymph node clearance. FP were cases with positive cytology at FNA cytology but were free of metastasis at axillary lymph node clearance. There were no false positives in this study. Sensitivity and NPV were 72%; specificity and PPV were 100%.

TP were the cases with evidence of metastatic disease on FNA cytology/CNB that had metastatic deposits proven at axillary lymph node clearance (n = 46). TN were those cases with negative cytology or histology for malignancy and were free of metastasis according to the gold standard SLNB (n = 28). FN were cases with negative cytology or histology for malignancy but at SLNB where found to have metastasis (n = 7). These patients then proceeded to have axillary lymph node clearance. FP were cases with positive cytology or histology and free of metastasis at axillary lymph node clearance; there were no false positives in our study. CNB was more sensitive than FNA cytology and was statistically significant (p = 0.006).

## Discussion

We demonstrated that patients in our series undergoing US-guided FNA cytology as a first line investigation are more likely to require a repeat procedure prior to their definitive surgery. Around 34.8% of patients that underwent this as a first line investigation required a repeat procedure compared to 2.63% of patients that had a US-guided CNB as their first line investigation. The use of US alone has a reported sensitivity of 61.4% (95% confidence intervals 50.7–71.2); as a result we focused on the combined accuracy of US with tissue sampling [6].

A study in 2012 reported a similar specificity and PPV of 100% (n = 210) for US-guided CNB which was similar to our findings, but reported a significantly lower sensitivity (52.4% versus 100%) [7]. This could be partly explained by differing tumour grades. A higher grade is more likely to be detected (sensitivities of 61% for grade 3 malignancies versus 35% for grade 1, p = 0.0146) [7]. However, the grading in our series was not recorded and perhaps this count could account for the lower sensitivity. In addition, differences in operatorʼs experience could also explain the lower sensitivity in their series. Higher sensitivities for CNB have been reported, a prospective study observed higher sensitivities for CNB versus FNA cytology (88.2% versus 72.5%, p = 0.008) with 100% specificity for both [6]. A significant difference between the sensitivity and NPV of CNB and FNA cytology were noted in our study. None of the 38 patients that had CNB sampling of their lymph nodes required a second operation (FP = 0). However, seven patients (7/43) that had negative cytology at FNA cytology had positive lymph nodes identified at SLNB and therefore required a second surgical procedure (p = 0.0066).

In contrast, a meta-analysis found no significant difference in sensitivity between the two (FNA cytology 72.2%, 95% CI 63.9–79.3 versus CNB 83.3%, 95% CI 70.0–91.4) [8]. However, the studies incorporated in the analysis had differing US criteria for identifying suspicious lymph node lesions, thus potentially introducing significant heterogeneity. These findings were supported in a later study which favoured FNA cytology for financial reasons [8]. However, high FP for FNA cytology have been identified as a potential pitfall, although our study did not reproduce these findings [9, 10]. Inadequate sampling of FNA cytology is another pitfall that has been identified [9, 11]. Repeated sampling and a lower sensitivity could result in inadequately treated breast cancer.

In 2014, a review (n = 10,934) reported the accuracy of preoperative US and CNB for axillary staging in invasive breast cancer on two meta-analyses [12]. The use of US alone (n=4313) gave a median sensitivity 61.4% (interquartile range [IQR] 51.2-79.4%) and median specificity of 82% (IQR, 76.9–89.0%). Further analysis (n = 2805) of the accuracy of CNB established a pooled sensitivity of 79.6% (95 % CI 74.1–84.2%) and a high pooled specificity of 98.3% (95% CI 97.2–99%). This study also reported a similar PPV to ours (100%, 95% CI 100–100%) but a lower NPV (67.4%, 95% CI 60–76.2%) [13]. In another meta-analysis, the combination of US with selective CNB (n=9212) reported a pool sensitivity of 50% (95% CI 43–57%) with a FN rate of 25% (95%CI 24–27%) [14]. However, it should be noted that there was significant heterogeneity amongst the studies, making meaningful comparisons difficult. Some of which is likely to be attributed to the fact that such procedures are highly operator dependent.

A study in 2016 looked at all cases with newly diagnosed ipsilateral primary breast cancer that underwent axillary US-guided biopsies in a two year period with outcomes compared to the final histopathology from SLNB or ANC. They found there was a correlation towards CNB but no statistically significant in favour of either technique [15]. Similarly, a recent review reported no absolute superiority of CNB over FNA cytology and stated that cytopathologist experience is likely to influence the reported differences in the procedures [16]. Indeed, this suggests that operator skill is likely to play a large role.

A retrospective analysis reported a sensitivity for CNB of 69.1% and specificity of 100% (n = 650); as a result, SLNB was avoided in 33% of patients [17]. CNB reduces the need for a second procedure which is beneficial to both the patient (reduced anxiety, reduced discomfort, fewer hospital visits) and the department.

In 2017, a meta-analysis (n = 1082 patients across 12 articles) reported a lower pooled sensitivity of 74% (95% CI 72–77%for FNA cytology compared to the pooled sensitivity of CNB (87%; 95% CI 84–88%). However, there was significant heterogeneity for both analyses (I^2^over 88%) implying that some caution should be used with interpreting the results. They reported similar specificities between CNB and FNA cytology (98%; 95% CI 96%–99%, I^2^= 76.2% versus 96%; 95% CI, 94–98%, I^2^ = 39.0%) [18]. Again, the high heterogeneity particularly for CNB results should be considered. Nevertheless, they reported that CNB has a superior sensitivity to FNA cytology.

There are limitations to our study as axillary US and tissue sampling are operator dependent techniques and this may have influenced our results. Secondly, tumour characteristics and patient demographics were not looked at in this study. The sensitivity for preoperative US-guided FNA cytology of abnormal lymph nodes in invasive ductal carcinoma has been shown to be high but not so for invasive lobular carcinoma (98.4% versus 53.6%, p<0.001, n = 142 axillae) [19]. This suggests that FNA cytology may not be a suitable method in all patients for preoperative axillary assessing in invasive lobular carcinoma. Therefore, these factors may have influenced the accuracy of US findings and node positive rates in our study. Lastly, our study focused on US guided tissue sampling techniques and hence only consecutive cancer patients who had US scans were included which may have introduced a selection bias.

## Conclusion

We found US-guided CNB to be a superior interventional technique when interrogating the axilla in our series. It is therefore recommended to perform a CNB as the first line investigation of potentially abnormal axillary lymph nodes, wherever possible, in order to reduce the likelihood of the patient requiring a second procedure whilst also improving sensitivity and NPV. Axillary US-guided CNB remains a valuable triage test for preoperative staging of the axilla as it helps to identify those patients unsuitable for SLNB. A large double-blind randomised controlled trial is needed for establishing whether any relationships exist between tumour characteristics and patient demographics and accuracy of US and lymph node sampling. Larger studies are also needed to quantify the role of the operatorʼs experience in performing these investigations with patients blinded to having either US guided CNB or FNA cytology.

## List Of Abbreviations

CNB: core needle biopsy
FNA: fine needle aspiration
PPV: positive predictive value
NPV: negative predictive value
US: ultrasound
TP: true positives
TN: true negatives
FN: false negatives
SLNB: sentinel lymph node biopsy
FP: false positives
ANC: axillary lymph node clearance.

## Conflicts of Interest

The authors report no conflicts of interest.

## Figures and Tables

**Table 1.** Diagnostic performance of CNB and FNA cytology.

Measure	CNB (%)	FNA cytology (%)
Sensitivity	100	72
Specificity	100	100
Negative predictive value	100	72
Positive predictive value	100	100

CNB: core needle biopsy, FNA: fine needle aspiration
